# Nationwide quality assurance of high-throughput diagnostic molecular testing during the SARS-CoV-2 pandemic: role of the Belgian National Reference Centre

**DOI:** 10.1186/s12985-024-02308-y

**Published:** 2024-02-10

**Authors:** Reile Janssen, Lize Cuypers, Lies Laenen, Els Keyaerts, Kurt Beuselinck, Sunita Janssenswillen, Bram Slechten, Jannes Bode, Elke Wollants, Kristel Van Laethem, Annabel Rector, Mandy Bloemen, Anke Sijmons, Nathalie de Schaetzen, Arnaud Capron, Kurt Van Baelen, Thierry Pascal, Céline Vermeiren, Fabrice Bureau, Jo Vandesompele, Pieter De Smet, Wouter Uten, Hugues Malonne, Pierre Kerkhofs, Jo De Cock, Veerle Matheeussen, Bruno Verhasselt, Laurent Gillet, Gautier Detry, Bertrand Bearzatto, Jonathan Degosserie, Coralie Henin, Gregor Pairoux, COVID-19 Genomics Belgium Consortium, Emmanuel André, Piet Maes, Guy Baele, Simon Dellicour, Lize Cuypers, Marc Van Ranst, Barney Potter, Samuel Hong, François E. Dufrasne, Guillaume Bayon-Vicente, Ruddy Wattiez, Carl Vael, Lynsey Berckmans, Philippe Selhorst, Kevin K. Ariën, Arnaud Marchant, Coralie Henin, Benoit Haerlingen, Ricardo De Mendonca, Marie-Luce Delforge, Sonia Van Dooren, Bruno Hinckel, Hideo Imamura, Toon Janssen, Ben Caljon, Oriane Soetens, Denis Piérard, Thomas Demuyser, Charlotte Michel, Olivier Vandenberg, Sigi van den Wijngaert, Giulia Zorzi, Jean Ruelle, Benoit Kabamba Mukadi, Jean-Luc Gala, Bertrand Bearzatto, Jérôme Ambroise, Philippe Van Lint, Walter Verstrepen, Reinout Naesens, Michael Peeters, Kate Bakelants, Sarah Denayer, Sofieke Klamer, Pascale Hilbert, Sylvain Brohée, Pierre-Emmanuel Léonard, Deniz Karadurmus, Jeremie Gras, Damien Féret, Barbara Lambert, Anne Vankeerberghen, Astrid Holderbeke, Hans De Beenhouwer, Lien Cattoir, Christine Lammens, Basil Britto Xavier, Marie Le Mercier, Jasmine Coppens, Veerle Matheeussen, Herman Goossens, Geert A. Martens, Koen Swaert, Frederik Van Hoecke, Dieter Desmet, Merijn Vanhee, Pierre Bogaerts, Jonathan Degosserie, Olivier Denis, Te-Din Huang, Dagmar Obbels, Hanne Valgaeren, Johan Frans, Annick Smismans, Paul-Emile Claus, Truus Goegebuer, Ann Lemmens, Bea Van den Poel, Sonja De Bock, Wim Laffut, Ellen Van Even, Jos Van Acker, Charlotte Verfaillie, Elke Vanlaere, Klara De Rauw, Brigitte Maes, Guy Froyen, Bert Cruys, Ellen Geerdens, Luc Waumans, Britt Van Meensel, Reinoud Cartuyvels, Severine Berden, Marijke Raymaekers, Bruno Verhasselt, Cécile Meex, Keith Durkin, Laurent Gillet, Maria Artesi, Marie-Pierre Hayette, Sébastien Bontems, Vincent Bours, Claire Gourzonès, Olivier Ek, Fabrice Bureau, Jorn Hellemans, Patrick Descheemaeker, Marijke Reynders, Piet Maes, Marc Van Ranst, Katrien Lagrou, Elisabeth Dequeker, Emmanuel André

**Affiliations:** 1 National Reference Centre for Respiratory Pathogens, Department of Laboratory Medicine, University Hospitals Leuven, 3000 Leuven, Belgium; 2 Laboratory of Clinical Microbiology, Department of Microbiology, Immunology and Transplantation, KU Leuven, 3000 Leuven, Belgium; 3 Federal Testing Platform COVID-19, Department of Laboratory Medicine, University Hospitals Leuven, 3000 Leuven, Belgium; 4 Laboratory of Clinical and Epidemiological Virology, Department of Microbiology, Immunology and Transplantation, Rega Institute for Medical Research, KU Leuven, 3000 Leuven, Belgium; 5 Quality of Laboratories Unit, Scientific Directorate of Biological Health Risks, Sciensano, 1000 Brussels, Belgium; 6 Janssen Pharmaceutica N.V, Johnson & Johnson, 2340 Beerse, Belgium; 7 GSK Vaccines, 1330 Rixensart, Belgium; 8 UCB Pharma, 1420 Braine L’Alleud, Belgium; 9 Laboratory of Cellular and Molecular Immunology, GIGA Institute, University of Liège, 4000 Liège, Belgium; 10 Biogazelle, a CellCarta Company, Technologiepark Zwijnaarde, 9052 Zwijnaarde, Belgium; 11 CliniSys|MIPS, 9000 Ghent, Belgium; 12 UgenTec N.V, 3500 Hasselt, Belgium; 13 Federal Agency for Medicines and Health Products (FAGG-AFMPS), 1210 Brussels, Belgium; 14 Department of Pharmacology, Pharmacotherapy and Pharmaceutical Care, Faculty of Pharmacy, Université Libre de Bruxelles, 1070 Brussels, Belgium; 15 Department of Biomedical Sciences, Namur Research Institute for Life Sciences, University of Namur, 5000 Namur, Belgium; 16 Federal Public Service Public Health, Safety of the Food Chain and the Environment, 1210 Brussels, Belgium; 17 National Institute for Health and Disability Insurance (RIZIV/INAMI), 1150 Brussels, Belgium; 18 Federal Testing Platform COVID-19, University Hospitals Antwerp, 2650 Edegem, Belgium; 19 Federal Testing Platform COVID-19, Department of Laboratory Medicine, Ghent University and Ghent University Hospital, 9000 Ghent, Belgium; 20 Federal Testing Platform COVID-19, University of Liège, 4000 Liège, Belgium; 21 Federal Testing Platform COVID-19, Laboratory of Clinical Biology, Pole Hospitalier Jolimont, 7100 La Louvière, Belgium; 22 Federal Testing Platform COVID-19, Centre Des Technologies Moléculaires Appliquées (CTMA), Institute of Experimental and Clinical Research (IREC), Cliniques Universitaires Saint-Luc and Université Catholique de Louvain (UCLouvain), 1200 Brussels, Belgium; 23 Federal Testing Platform COVID-19, Department of Laboratory Medicine, CHU UCL Namur, 5530 Yvoir, Belgium; 24 Federal Testing Platform COVID-19, Université Libre de Bruxelles, 1070 Brussels, Belgium; 25 Biomedical Quality Assurance Research Unit, Department of Public Health and Primary Care, University of Leuven, 3000 Leuven, Belgium

**Keywords:** SARS-CoV-2, COVID-19, Belgium, Quality assurance, High-throughput testing, National reference centre

## Abstract

Since the onset of the coronavirus disease (COVID-19) pandemic in Belgium, UZ/KU Leuven has played a crucial role as the National Reference Centre (NRC) for respiratory pathogens, to be the first Belgian laboratory to develop and implement laboratory developed diagnostic assays for SARS-CoV-2 (severe acute respiratory syndrome coronavirus 2) and later to assess the quality of commercial kits. To meet the growing demand for decentralised testing, both clinical laboratories and government-supported high-throughput platforms were gradually deployed across Belgium. Consequently, the role of the NRC transitioned from a specialised testing laboratory to strengthening capacity and coordinating quality assurance. Here, we outline the measures taken by the NRC, the national public health institute Sciensano and the executing clinical laboratories to ensure effective quality management of molecular testing throughout the initial two years of the pandemic (March 2020 to March 2022).

## Introduction

For more than a decade, a network of national reference centres (NRCs) for human microbiology has been set up in Belgium. Altogether, this network is in charge of the surveillance of infectious diseases and, depending on each specific pathogen, is responsible for first line diagnostics, second line specialised tests or for supporting quality assurance when first line tests are decentralised in multiple clinical laboratories. In addition, this network coordinates the public health surveillance and responsibilities are defined in detail by a Royal Decree [1]. The main responsibility of a NRC is to support health care workers, clinical laboratories and epidemiologists by contributing to the diagnosis and further characterisation of specific infections. In addition to providing technical assistance to clinical laboratories, NRCs are expected to support surveillance programs and provide technical advice to the government in the context of public health threats such as emerging infections, outbreak settings and epidemics.

With the emergence of SARS-CoV-2, the role of the NRC for SARS-CoV-2 was integrated into the existing NRC for respiratory pathogens hosted by the laboratory of the University Hospitals Leuven (UZ Leuven), which is ISO 15189 accredited [1]. As part of its NRC mandate, UZ Leuven was the first laboratory in Belgium to evaluate, develop and implement multiple SARS-CoV-2 diagnostic assays. In addition, the NRC actively contributed to the roll-out of diagnostic tests throughout the country. Although testing capacity increased rapidly from March 2020 as a result of a constantly increasing number of clinical laboratories implementing diagnostic tests, the demand for testing could not be met during the first wave of infections [2]. To allow for a rapid temporary expansion of the COVID-19 test capacity, a national testing platform involving pharmaceutical industry and academic partners was set up in April 2020 by the Belgian Federal Government and its Federal Agency for Medicines and Health Products (FAMHP). This temporary structure was replaced from October 2020 on by eight federal testing platforms, each co-led by a university and an accredited clinical laboratory. Overall, these ad hoc temporary laboratories have processed more than 6 million respiratory samples over two years. The rapid scale-up of testing as well as the implementation of high-throughput platforms involved major challenges such as the recruitment and training of personnel, ensuring continuous access to reagents, consumables and sampling material, deployment of high-throughput workflows and infrastructure, and finally establishing novel pre- and post-analytical workflows [3–6].

Inevitably, the unprecedented scale of the COVID-19 pandemic and the important challenges also specifically impacted the NRC, which needed to ensure that its legal tasks and obligations were met despite the unprecedented context [1]. Deploying these tasks also implied establishing strong collaborations with Sciensano (the Belgian institute for public health), the government and the various groups that were set up during of the pandemic. In this article, we review the steps taken by the SARS-CoV-2 NRC to build and maintain a quality assurance system covering molecular testing during the first two years of the pandemic (March 2020—March 2022). A retrospective evaluation of measures taken can be of use in establishing or adjusting policies, operational plans and resources for the rapid scale-up of testing capacity for a potential future pandemic.

## Evolution of SARS-CoV-2 testing at the NRC UZ/KU Leuven

Prior to the SARS-CoV-2 pandemic, the NRC for Respiratory pathogens had validated a lab-developed test (LDT) for routine diagnosis of upper and lower respiratory tract infections. This respiratory panel assay had been established since 2016 and consists of 12 real-time multiplex PCRs detecting twenty-two viruses (i.e., influenza A, influenza B, parainfluenza virus (PIV)-1, PIV-2, PIV-3, PIV-4, respiratory syncytial virus (RSV) A and B, rhino-enterovirus, enterovirus D68, herpes simplex virus (HSV)-1, HSV-2, human metapneumovirus, adenovirus, bocavirus, parechovirus, human coronavirus (HCoV) 229E, HCoV HKU-1, HCoV NL63, HCoV OC43, MERS-CoV, SARS-CoV-1 and cytomegalovirus (CMV)), in addition to several bacteria and fungi (i.e., Mycoplasma pneumoniae, Coxiella burnetii, Chlamydia pneumoniae, Chlamydia psittaci, Streptococcus pneumoniae, Legionella pneumophila and Pneumocystis jirovecii) [7]. After the first SARS-CoV-2 genome became available on January 10, 2020 [8], this respiratory panel was slightly modified to be able to detect all sarbecoviruses, including SARS-CoV-2 (Table 1A). Prior to the wide circulation of SARS-CoV-2 in Belgium, the use of a syndromic approach allowed comprehensive screening of all patients with flu-like symptoms or travellers returning from affected countries [9]. Therefore, using a broad respiratory panel was not only the fastest way to meet the urgent demand for COVID-19 testing, it also enabled to rule out influenza which was in an epidemic phase in Asia at the time. From the moment evidence of active virus circulation in the country could be gathered in early February 2020, the need for testing increased and therefore the NRC developed a dual target SARS-CoV-2 LDT qPCR (quantitative polymerase chain reaction) on the Open Access functionality of the Panther Fusion sample-to-result (S2R) robotic system (Hologic, Marlborough, Massachusetts, United States). The use of a continuous random-access platform enabled to provide fast turn-around times (TATs) without the need for sample batching. Samples of patients with a differential diagnosis of influenza-like illness were simultaneously tested with the FluA/B/RSV assay on Panther Fusion [10].

**Table 1:** Overview of the assays used for SARS-CoV-2 detection, antibody detection and genotyping at the NRC UZ/KU Leuven and at the UZ Leuven hospital. The assays currently in use are marked in bold

Assay	Assay description		Device type		Instrument (manufacturer)			SARS-CoV-2 targets		Other targets		Instrument set-up		Instrument capacity (tests/24 h)		TAT (min)	Implementation date
**A**. Overview of assays used for SARS-CoV-2 detection																	
**LDT Respiratory panel**	NA extraction + qPCR		LDT		eMAG (BioMérieux) + QuantStudio Dx (TMO)			ORF1ab		IC and 28 other pathogens*		Open system (batch)		112		300	16–01-2020
LDT SARS-CoV-2 eMAG/ QuantStudio	NA extraction + qPCR		LDT		eMAG (BioMérieux) + QuantStudio Dx (TMO)			E		IC		Open system (batch)		576		240	31–01-2020
LDT SARS-CoV-2 Panther Fusion	NA extraction + qPCR		LDT		Panther Fusion (Hologic)			E, ORF1ab		IC		S2R system		750		150	17–02-2020
LDT SARS-CoV-2 KingFisher/ QuantStudio	NA extraction + qPCR		LDT		KingFisher Flex (TMO) + QuantStudio Dx (TMO)			E		IC		Open system (batch)		2160		240	04–03-2020
Xpert Xpress SARS-CoV-2	NA extraction + qPCR		CE-IVD (FDA EUA)		GeneXpert IV (Cepheid)			N2, E		IC		S2R system		96		60	07–05-2020
Aptima SARS-CoV-2 Assay	NA extraction + TMA		CE-IVD (FDA EUA)		Panther System (Hologic)			ORF1ab		IC		S2R system		750		210	08–06-2020
**Alinity m SARS-CoV-2 Assay**	NA extraction + qPCR		CE-IVD (FDA EUA)		Alinity m (Abbott)			N, RdRP		IC		S2R system		1000		140	14–10-2020
Xpert Xpress SARS-CoV-2/ Flu/ RSV	NA extraction + qPCR		CE-IVD (FDA EUA)		GeneXpert IV (Cepheid)			N2, E		IC; M1, PB2, PA gene (Influenza A); M1, NS1 gene (Influenza B); N gene (RSV)		S2R system		160		36	12–01-2021
Panbio COVID-19 Ag Rapid Test Device	Rapid chromatographic immunoassay		CE-IVD(FDA EUA)		Abbott			N protein		IC		N/A		N/A		15	26–01-2021
MagMAX Viral/Pathogen II Nucleic Acid Isolation Kit + TaqPath COVID-19 CE-IVD RT-PCR kit	NA extraction + qPCR		CE-IVD (FDA EUA)		Freedom EVO 150 (Tecan) + KingFisher Flex (TMO) + QuantStudio 7 Flex (TMO)			N, ORF1ab, S		IC		Open system (batch)		864		150	09–02-2021
**Cobas SARS-CoV-2**	NA extraction + qPCR		CE-IVD (FDA EUA)		Cobas Liat (Roche)			N, ORF1ab		IC		S2R system		72		20	17–10-2021
**Cobas SARS-CoV-2 & Influenza A/B**	NA extraction + qPCR		CE-IVD (FDA EUA)		Cobas Liat (Roche)			N, ORF1ab		IC; M1 gene (Influenza A); NS1 gene (Influenza B)		S2R system		72		20	10–12-2021
**Xpert Xpress SARS-CoV-2/ Flu/ RSV plus**	NA extraction + qPCR		CE-IVD (FDA EUA)		GeneXpert IV (Cepheid)			N2, E, RdRP		IC; M1, PB2, PA gene (Influenza A); M1, NS1 gene (Influenza B); N gene (RSV)		S2R system		160		36	21–01-2022
**Alinity m Resp-4-plex assay**	NA extraction + qPCR		CE-IVD (FDA EUA)		Alinity m (Abbott)			N, RdRP		IC; M1 gene (Influenza A); NS1 gene (Influenza B); M gene (RSV)		S2R system		1040		140	31–03-2022
**Xpert Xpress SARS-CoV-2 plus**	NA extraction + qPCR		CE-IVD (FDA EUA)		GeneXpert IV (Cepheid)			N2, E, RdRP		IC		S2R system		192		30	08–09-2022
**B.** Overview of assays used for SARS-CoV-2 antibody detection																	
SARS-CoV-2 IgG		CMIA		CE-IVD (FDA EUA)		Architect i2000 SR (Abbott)	N		N/A		S2R System		4800		30		29–04-2020
SARS-CoV-2 IgG II Quant		CMIA		CE-IVD (FDA EUA)		Architect i2000 SR (Abbott)	S		N/A		S2R system		4800		30		07–04-2021
**SARS-CoV-2 IgG**		CMIA		CE-IVD (FDA EUA)		Alinity I (Abbott)	N		N/A		S2R system		4800		30		08–02-2023
**SARS-CoV-2 IgG II Quant**		CMIA		CE-IVD (FDA EUA)		Alinity i (Abbott)	S		N/A		S2R system		4800		30		08–02-2023
**C.** Overview of assays used for SARS-CoV-2 genotyping																	
**SARS-CoV-2 WGS**		NA extraction + WGS		RUO		Freedom EVO 150 (Tecan) + KingFisher Flex (TMO) + MinION/ GridION (ONT)	complete SARS-CoV-2 genome		N/A		Open system (batch)		480		2880		03–02-2020
MagMAX Viral/ Pathogen II Nucleic Acid Isolation kit + TaqMan SARS-CoV-2 Mutation Research Panel		NA extraction + qPCR		RUO		Freedom EVO 150 (Tecan) + KingFisher Flex (TMO) + QuantStudio 5 (TMO)	S gene: 417KNT, 452LRQ, 484EKQ, 501NYT, 681PHR,		N/A		Open system (batch)		768		210		07–01-2021

CMIA (chemiluminescent microparticle immunoassay), EUA (emergency use authorization), FDA (Food and Drug Administration), IC (internal control), IVD (in vitro diagnostic medical device), LDT (lab-developed test), N/A (not applicable), NA (nucleic acid), ONT (Oxford Nanopore Technologies), RUO (Research Use Only), S2R (sample to result), TAT (turn-around-time), TMA (transcription-mediated amplification), TMO (Thermo Fisher Scientific), WGS (Whole Genome Sequencing). (*) Influenza A, influenza B, parainfluenza virus (PIV)-1, PIV-2, PIV-3, PIV-4, respiratory syncytial virus (RSV) A and B rhino-enterovirus, enterovirus D68, herpes simplex virus (HSV)-1, HSV-2, human metapneumovirus, adenovirus, bocavirus, parechovirus, human coronavirus (HCoV) 229E, HCoV HKU-1, HCoV NL63, HCoV OC43, MERS-CoV, SARS-CoV-1, cytomegalovirus (CMV), *Mycoplasma pneumoniae, Coxiella burnetii, Chlamydia pneumoniae, Chlamydia psittaci, Streptococcus pneumoniae, Legionella pneumophila* and *Pneumocystis jirovecii*.

By March 2020, laboratories were confronted with worldwide shortages in reagent supplies since manufacturers were not able to keep up with an exponential increase in demand. To compensate these shortages and to optimise testing capacity, additional molecular SARS-CoV-2 LDT’s were implemented on open platforms, and nucleic acid (NA) extraction steps where separated from the qPCR analysis to allow more flexibility in analytical workflows (Table 1A) [2]. This strategy nevertheless required batching of samples, inevitably associated with a slight increase in TAT, particularly when lower number of samples were received, such as during night shifts. NA extraction was initially performed on easyMAG and eMAG instruments (BioMérieux, Marcy-l'Étoile, France) enabling simultaneous processing of 24 and 48 samples, respectively. In a later phase, these instruments were replaced by KingFisher Flex extraction robots (Thermo Fisher Scientific, Waltham, Massachusetts, United States) permitting NA extraction in 96-well plates, thereby further increasing capacity. SARS-CoV-2 RNA was detected by qPCR on QuantStudio Dx using primers and probes adapted from Corman et al. [11].

On March 12, 2020, one day after the World Health Organization declared the COVID-19 outbreak a worldwide pandemic, SARS-CoV-2 diagnostics entered a new phase as the U.S. Food and Drug Administration (FDA) issued its first Emergency Use Authorization for an IVD molecular diagnostic test originating from a commercial manufacturer. This facilitated further implementation of diagnostic testing in clinical laboratories, thereby increasing the Belgian test capacity. At the NRC, LDT’s were sequentially replaced by CE-IVD alternatives, starting with the implementation of the Xpert Xpress SARS-CoV-2 assay, an automated test based on RT-PCR on the GeneXpert IV (Cepheid).

The setup of an automated open platform incorporating sample and qPCR preparation by EVO 150 liquid handlers (Tecan, Männedorf, Switzerland), NA extraction on KingFisher Flex (Thermo Fisher Scientific, Waltham, Massachusetts, United States), qPCR on QuantStudio 7 Flex using the TaqPath COVID‑19 CE‑IVD RT‑PCR Kit (Thermo Fisher Scientific, Waltham, Massachusetts, United States), and FastFinder Analysis software (UgenTec) to analyse PCR data and the SARS-CoV-2 assay on an Alinity m random access S2R system (Abbott, Chicago, Illinois, United States) ensured further upscaling of testing capacity. In light of the need to differentiate between past and recent infections, and in order to relay detailed information about the amount of SARS-CoV-2 genetic material present in each sample, quantitative reporting was set up for both high throughput assays.

Furthermore, IgG anti-N and anti-S antibody detection by chemiluminescent microparticle immunoassay (CMIA) on Architect i2000SR (Abbott, Chicago, Illinois, United States) was introduced (Table 1B), providing the ability to differentiate between a natural and a Spike protein-based vaccine-induced adaptive immune response. The clinical utility of these tests and their use declined over the sequential waves of infections, as the immunity of the population increased.

In addition to the high throughput qPCR platforms, rapid PCR tests, either at the laboratory or as a point-of-care test, enabled rapid analysis of samples in particular contexts such as the emergency unit or the maternity department. For this purpose, the Xpert Xpress SARS-CoV-2 and Xpert Xpress SARS/Flu/RSV on GeneXpert (Cepheid, Sunnyvale, California, United States) and the Cobas SARS-CoV-2 assay and Cobas SARS-CoV-2 & Influenza A/B Assay on Cobas Liat (Roche, Basel, Switzerland) were validated and implemented within UZ Leuven. Additionally, rapid antigen tests were introduced in the maternity and oncology department in January 2021, using the Panbio COVID-19 Ag rapid test device (Abbott, Chicago, Illinois, United States).

The first case of COVID-19 in Belgium was detected on February 3, 2020, and was confirmed on the same day through whole genome sequencing (WGS) using the MinION platform (Oxford Nanopore Technologies, Oxford, United Kingdom) and the ARTIC network v.1 primer pools (Table 1C) [12]. Subsequently, WGS has been consistently performed on a small subset of PCR positive samples, either to address complex transmission issues or for surveillance purposes. In early 2021, heightened concerns surrounding the circulation of Alpha, Beta and Gamma variants of concern (VOCs) prompted the initiation of a comprehensive National Genomic Surveillance Initiative, expanding Belgium’s WGS capacity [13]. In addition to WGS, a multiplex qPCR genotyping assay utilizing the TaqMan SARS-CoV-2 Mutation Assays (Thermo Fisher Scientific, Waltham, Massachusetts, United States) was implemented shortly after the emergence of the first VOC (Alpha) in January 2021. This assay enables the identification of specific mutations of concern facilitating the discrimination of co-circulating VOCs with short TATs. The latter method was discontinued in January 2022, due to the increasing genetic diversification of SARS-CoV-2.

## Increasing the Belgian testing capacity while assuring high quality test results

### Supporting routine diagnostics for COVID-19 in the clinical laboratories

Following the introduction and subsequent community transmission of SARS-CoV-2 in Belgium, there was a need to roll-out large-scale and decentralised testing at the national level. At that time, many clinical laboratories did not have experience in conducting a wide arsenal of molecular assays. Further, considering that SARS-CoV-2 was a new virus for which knowledge, experience and reference material was limited at the start of the pandemic, the NRC had to develop strategies to compensate for existing limitations and had to organise technical support for starting laboratories, while needing to compensate unmet demand for testing with its own existing diagnostic platforms.

Considering the multiplicity of diagnostic assays deployed throughout the large network of clinical laboratories and the need to thoroughly support the validation in each particular setting, the NRC provided positive reference material to each requesting laboratory, as well as additional technical support and advice through close contact with the medical and scientific staff of each laboratory. SARS-CoV-2 positive control material was prepared by culturing a nasopharyngeal swab sample, originating from the first SARS-CoV-2 positive patient diagnosed in Belgium, according to the protocol as described in Cuypers et al. [14]. This heat-inactivated control material was broadly shared across Belgium (approval of UZ/KU Leuven Ethics committee for research (S64181)) accompanied with a letter containing the determined viral load, Nextclade [15, 16] and Pangolin [17, 18] classification and GISAID-ID [19] (EPI_ISL_407976) in addition to the detected amino acid changes.

**Fig. 1:**
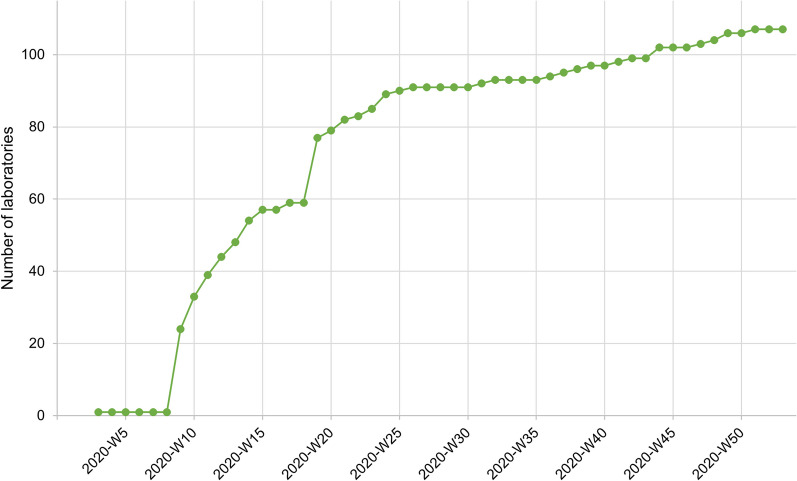
Evolution of Belgian laboratories recognised in 2020 to perform SARS-CoV-2 PCR-testing. Up until week 8, solely the NRC conducted COVID-19 tests. The Belgian testing capacity expanded gradually from March 1, 2020 on, as clinical laboratories were granted permission by Sciensano to initiate diagnostic testing. By the end of the year, 107 laboratories were performing SARS-CoV-2 PCR tests

In Belgium, all clinical biology laboratories are required to work under a quality management system (QMS) and the molecular tests must be included in the scope of an ISO 15189 accreditation [20, 21]. The Belgian accreditation body (BELAC) and Sciensano are responsible for the accreditation and licencing respectively of these laboratories and the monitoring of their QMS [22]. However, for a clinical laboratory to be officially recognised to perform COVID-19 testing and thus receive reimbursement of costs for diagnostic tests from RIZIV/INAMI, the federal public body of social security in Belgium, a number of predetermined conditions had to be met. First, laboratories had to summarise information regarding fulfilment of the predefined conditions to be met by their quality system as described in the Clinical Biology Code of Practice [23] regarding their mandatory ISO 15189 accreditation [20, 21] as well as regarding organisational aspects (i.e., testing capacity, kits and equipment, laboratory technicians and medical staff, …), which was evaluated by the quality service unit of Sciensano. Additionally, each laboratory was obligated to participate to the external quality control (QC) program for SARS-CoV-2 organised by Quality Control for Molecular Diagnostics (QCMD) in 2020 [24]. As a last criterion, laboratories were obliged to send five positive samples to the NRC for confirmatory testing. Only when these test results could be confirmed by the NRC, recognition of the laboratory to officially perform COVID-19 tests was granted by Sciensano. Figure 1 gives an overview of the number of laboratories in Belgium that supported the test capacity after meeting all the above criteria as imposed by Sciensano. Up to week 8, SARS-CoV-2 PCR testing was only performed at the NRC, whereafter other laboratories were granted permission to start testing and testing capacity began to expand gradually.

To provide regular updates in a rapidly evolving field, the NRC organised frequent online information sessions with all clinical laboratories. These sessions covered a wide variety of topics including testing strategy, data flow, evaluation of assays and technical issues (Table 2). During these information sessions, laboratories had the opportunity to raise questions regarding problems or bottlenecks, as well as exchange experiences and receive input from other laboratories, the NRC, Sciensano or RIZIV/INAMI. On average, more than 100 participants attended each of these information sessions, which were organised until June 2022.

**Table 2:** Overview of topics discussed during the weekly to monthly COVID-19 testing information sessions organised by the NRC UZ/KU Leuven throughout the course of the pandemic

Dates	Frequency	Categories of topics on the agenda			
		Testing strategy and guidelines	Data flow	Evaluation of assays	Bottlenecks in testing
12/03/2020 – 14/05/2020	Weekly	X	X	X	X
14/05/2020 – 10/07/2020	Bi-weekly	X	X	X	X
10/07/2020 – 02/06/2022	Monthly	X	X	X	

### Establishing high-volume testing platforms to rapidly expand the Belgian testing capacity

#### National testing platform

Despite the rapid implementation of SARS-CoV-2 testing by existing clinical laboratories and the subsequent increase in testing capacity, the latter remained below testing demand during the first wave of cases that Belgium experienced. To allow for a rapid temporary expansion of testing capacity, the Belgian Federal Government and its FAMHP announced the establishment of a national consortium for the upscaling of SARS-CoV-2 PCR testing, with a target capacity of 10,000 samples per day [25]. The setup of this so-called national testing platform involved pharmaceutical and biotechnological industry partners as well as academic partners, hereafter referred to as subcontracting (SC) laboratories, who performed PCR tests under the supervision of the NRC (the contracting laboratory) and Sciensano. All universities, research centres and industrial partners outside the partners of the consortium were called upon by the Belgian government to make equipment, laboratory space, staff and reagents available for the laboratories of the national testing platform, for a period of 6 months [26].

To limit SC laboratories’ access to patient data and facilitate sample traceability, patient identification was not labelled on the tube, but samples were collected in unique pre-barcoded sample tubes. Sampling was carried out at various collection sites across Belgium (e.g., test centres, nursing homes, care facilities), where the test prescription (prescriber and patient data) was linked to the sample tube through a government-established hub. A minimum of 65 sampling centres were set up throughout the country. The samples were then transferred to two central logistics centres where samples were batched and NRC QC samples were added as described in Van Vooren et al. [26], whereafter samples were dispatched among five SC testing laboratories. The latter reported results through the government health portal and only the requesting physician had access to the individual test results. Due to sample transportation and logistics, the SC laboratories had response times between 24 and 36 h, from sample collection to result, for more than 50% of the samples (data not shown).

Similar to what was implemented for clinical laboratories, each SC laboratory received heat-inactivated SARS-CoV-2 viral stock for initial validation of their SARS-CoV-2 assays and staff training, whereafter NRC and Sciensano revised descriptions of testing procedures for each laboratory. Additionally, before diagnostic testing could commence, each SC laboratory was provided with a blind qualification panel to evaluate their respective performance standards. Four SC labs received a randomised panel consisting of residual material of nasopharyngeal swab samples collected in universal transport medium (UTM, COPAN Diagnostics Inc. Brescia, Italy), containing 43 samples which tested positive for SARS-CoV-2 at the NRC using LDT SARS-CoV-2 eMAG/QuantStudio or LDT SARS-CoV-2 KingFisher/QuantStudio, with viral loads ranging from < 3.0 log RNA copies/mL to > 7.0 log RNA copies/mL. To evaluate analytical specificity, the panel included 11 samples which tested negative for SARS-CoV-2, but positive for other common respiratory pathogens (i.e., influenza A, RSV, enterovirus, rhinovirus, parechovirus, human metapneumovirus, HSV-1, adenovirus, CMV, Streptococcus pneumoniae) and 8 SARS-CoV-2 negative samples, but positive for HCoV NL63, HCoV 229E, HCoV OC43 or HCoV HKU-1. Additionally, samples were flanked by 32 blank samples containing UTM to ascertain the presence of cross-contamination. A fifth SC lab had already tested a randomised panel from the NRC, containing 40 SARS-CoV-2 positive samples (< 3.0 log to > 7.0 log RNA copies/mL) and 15 negative samples, as it provided regional support to a clinical laboratory. The latter laboratory tested an additional panel containing 25 samples before starting its function within the national platform. Since they used an assay for which sufficient analytical specificity data was already available, the inclusion of potentially cross-reactive samples was not deemed necessary. Based upon initial results of all tested panels, one SC lab decided to change NA extraction kits, after which a new randomised panel was tested.

After meeting the agreed upon testing requirements based on Rabenau et al. [27], SC laboratories started diagnostic testing. The available capacity of each SC laboratory was further scaled up by implementing an automated high-throughput workflow using standardised sample collection tubes with virus inactivating transport medium and liquid handlers for sample processing. For each change in the laboratory workflow, a cross-validation was performed using the original workflow as reference method. NRC and Sciensano revised all adjusted laboratory procedures and cross-validation results prior to diagnostic implementation.

During the summer period of 2020 following the first wave, several SC laboratories had a reduced testing capacity as a result of annual leave of employees. Therefore, additional test laboratories located in Germany and France, obligated to meet the same requirements as the Belgian SC laboratories, were also temporarily called upon to contribute to the Belgian test capacity. From the start of the set-up of the national testing platform until May 16, 2020, all involving parties met daily to forecast the number of samples and evaluate the distribution of samples. Additionally, further optimalisation of the process, non-conformities (NCs) and issues were discussed. This frequency shifted to meetings only on weekdays until June 5, 2020, whereafter the status calls were organised on Monday, Wednesday and Friday. In addition, until July 5, 2020, a technical meeting was organised twice a week with members of the SC laboratories and the NRC, to discuss technical and analytical issues and share experiences on day-to-day operations.

**Fig. 2:**
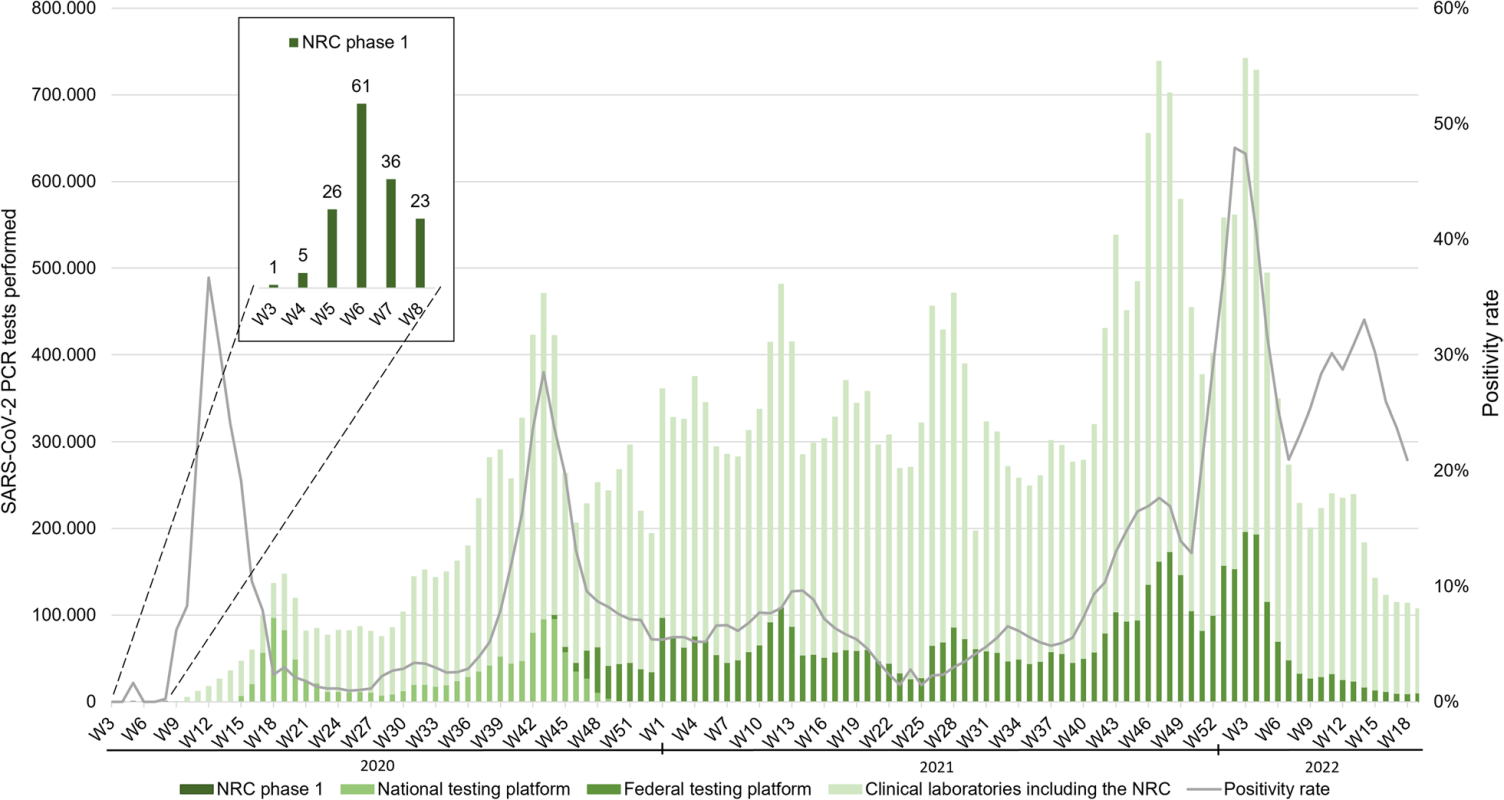
Weekly number of SARS-CoV-2 PCR tests performed in Belgium during the first two years of the pandemic, associated with the positivity rate. Up until week 8, PCR tests were only performed in the clinical laboratory of the NRC, after which other clinical laboratories were granted permission to perform PCR tests (see Fig. 1). In week 15, the national testing platform was founded to support the clinical laboratories, this initiative was replaced by the federal testing platform from October 2020 on

To enable daily monitoring and maintenance of high-quality standards for tests performed in the SC laboratories, a continuous proficiency testing program was set up by the NRC by using SARS-CoV-2 positive (POS), weakly positive (LOPOS) and negative QC samples, which were completely blinded for the SC laboratories as they were prepared in the standardised pre-barcoded sampling tubes and registered in an identical manner to clinical samples. All NRC QC samples were prepared, registered, distributed and evaluated as described in Van Vooren et al. [26] and results were reported daily to the SC laboratories and Sciensano. Over a seven-month period, the SC laboratories of the national testing platform collectively tested a total number of 1.3 million clinical samples (Fig. 2). Additionally, 5,562 NRC QC samples were tested of which 92 (1.65%) generated a discordant result. Failed POS (11/1,436 or 0.77%) and LOPOS (39/1,385 or 2.82%) QC samples could most often be traced back to a technical issue either during the extraction process or during the PCR set up, such as a plate swap or 180° plate rotation. For all 2,741 negative QC samples tested, 42 were falsely reported as positive (1.53%) due to cross-contamination, either during primary sample transfer or during PCR set up. Failed QC samples led to repetition of the affected run and a follow-up meeting was scheduled with the NRC and Sciensano to discuss the corrective and preventive actions, thereby improving the laboratory process in order to eliminate the cause of NCs. Whenever clinical samples showed discrepant results after retesting, results were corrected, and patients were informed via clinical biologists affiliated to the NRC.

Overall, despite limited experience with the rapid roll-out of high-throughput testing, a worldwide shortage in reagents and local differences in staffing and infrastructures, all Belgian laboratories succeeded in performing SARS-CoV-2 PCR tests on a large scale during the first months of the pandemic. However, support of the industry and academic partners, through the provision of personnel and infrastructure, was essential to quickly expand the national testing capacity and compensate for the gradual roll-out of conventional clinical laboratories. With the support of several external IT companies, a virtual laboratory with a unified Laboratory Information System was launched, allowing streamlined reporting of test results to patients and physicians, while safe-guarding anonymity of patient data from non-clinical testing laboratories [26].

#### Federal testing platform

After the summer period of 2020, as industrial partners of the national testing platform were slowly recommencing their day-to-day operations that were put on hold due to the nationwide lockdown, a novel consortium had to be created to always guarantee a high testing capacity. Therefore in October 2020, eight federal testing platforms, each driven by a collaboration between a university and an accredited clinical partner, were established to perform SARS-CoV-2 PCR tests on a large scale. It was anticipated for each laboratory to exclusively process samples collected in standardised pre-barcoded sample collection material containing virus inactivating medium and to report results within 24 h after sample collection. Further, each laboratory was asked to be able to increase their testing capacity up to 7,000 samples per day. The federal testing laboratories processed samples using the same workflow, since the government provided them with identical devices, consumables, reagents and software to analyse PCR data. Reagents and consumables used by the federal platform laboratories were selected through public tenders [28, 29], for which the NRC granted advice regarding drafting the technical specifications, created evaluation panels and consolidated panel results to evaluate the public procurement. Initially, all samples for the federal platform laboratories were collected in DNA/RNA Shield (Zymo Research, Europe GmbH, Baden-Württemberg, Germany), however, after all laboratories had carried out a thorough validation, a switch to sample collection in InActiv Blue medium (InActiv Blue, Beernem, Belgium) was made in September 2021.

Since the federal platform laboratories were considered an extension of an accredited clinical biology laboratory, they were working within the QMS and under the supervision of their partner clinical laboratory. On top of that, they had to meet specific quality requirements as set up by Sciensano and the NRC, as described in the consortium agreement. Therefore, prior to being allowed to test clinical samples, they were audited by Sciensano to verify these specific quality requirements. In addition, they had to successfully pass a qualification panel, created by the NRC, to evaluate their respective performance standards. Each randomised panel consisted of 91 samples of which 52 samples were positive for SARS-CoV-2 (with viral loads ranging from < 3.0 log RNA copies/mL to > 7.0 log RNA copies/mL) and 39 SARS-CoV-2 negative samples containing only the virus inactivating liquid DNA/RNA Shield (Zymo Research Europe GmbH, Baden-Württemberg, Germany). To assess repeatability, four positive samples were added in triplicate. All positive samples were prepared by diluting residual material of a strongly SARS-CoV-2 positive nasopharyngeal swab sample in tubes containing virus inactivating medium. To determine reference Cq values, one panel was tested at the NRC using LDT SARS-CoV-2 KingFisher/QuantStudio (Table 1A). Since one federal platform laboratory was already active as of March 2020 as SC laboratory and reported results consistent with previous NRC qualification panels and daily NRC QC samples, they were exempted from testing an additional qualification panel. The other seven laboratories passed the minimum criteria of the qualification panel, however minor differences in sensitivity between the laboratories were observed. Qualification panel results were consolidated in a report and evaluated for each laboratory during its ‘start-up’ audit, executed by Sciensano. Following this evaluation, all federal platform laboratories were granted permission to start testing clinical samples.

Given the importance of quality assurance, each clinical laboratory partner was obliged to add a panel of mock clinical samples, consisting of one SARS-CoV-2 positive, one weakly SARS-CoV-2 positive and three negative samples, to the clinical samples of the federal platform laboratory at least twice a week. If the weekly average number of samples tested in the federal platform laboratory exceeded 21,000, an additional control panel had to be added for every extra 21,000 samples tested. The partner clinical laboratory was also responsible for monitoring quality and response time and providing technical support where needed. In addition, each federal platform laboratory was required to monthly organise a quality, technical and logistics meeting with staff members of the clinical and platform laboratory, Sciensano and the NRC. During these mandatory Q-meetings results of the mock clinical control panels, (a selection of) all registered NCs, internal quality control (iQC) results, TAT, staffing and capacity planning, the organisation of additional validation and completion of validation reports, and the revision and need for additional procedures were evaluated and critically discussed.

To minimise logistic delays, the federal platform laboratories were geographically dispersed across the country, with three laboratories located in Flanders, three laboratories in the Walloon region and two laboratories based in the Brussels-Capital Region. Each federal platform laboratory collaborated with multiple testing centres and samples were transported directly from the sampling location to the laboratory, a predetermined time period of maximum 8 h was considered for transport and pre-analytical handling of the samples. As the laboratories were required to report results within a time frame of 24 h after sampling, a 16-h time period was established for the analytical testing and reporting of the samples. TATs were calculated weekly for each platform by Sciensano and reported to the laboratories, any discrepancies were discussed during the platform’s Q-meeting. Given that samples were transported directly to the laboratories, the pre-analytical TAT (from sampling to registration in the lab) reduced when the national platform was replaced with the federal platform, enabling the majority of samples receiving a result within 24 h after sampling [9]. To discuss topics such as testing strategy, materials, equipment, logistics, result reporting or general issues, a status-call was organised once a week with partners of each federal platform laboratory, logistic partners, the government’s task force, Sciensano and the NRC.

To enable an objective assessment of the quality of results generated by the federal platform laboratories, the NRC and Sciensano had set up an external quality assurance (EQA)-scheme. For each round, eight identical panels were prepared in the standardised pre-barcoded tubes and to all samples a nasopharyngeal swab was added, making them visually indistinguishable from clinical samples. In February 2021, a first EQA panel was distributed, consisting of 22 EQA samples of which 11 SARS-CoV-2 positive (viral loads ranging from 494 to 1.6 × 10^8^ RNA copies/mL) and 11 blank control samples, additionally three educative EQA samples with viral loads close to the analytical limit of detection (27 to 279 RNA copies/mL—no scoring) were added. Criteria for excellent performance were to correctly detect (qualitative result) all 22 EQA samples and to report all results within 16 h after reception, as required by the consortium agreement. All platform laboratories (8/8; 100%) obtained the maximum score for qualitative detection of the SARS-CoV-2 virus. Six laboratories (6/8; 75%) were able to correctly identify the three educative EQA samples. Two laboratories reported one or two samples as negative, it was known from the initial validation by the setup of the platforms that not all platforms could test with equal sensitivity. Regarding response time, four laboratories reported their results within the foreseen time period, the other four laboratories reported results outside of the predetermined TAT, due to confusion since transport of the EQA samples was performed by a private courier not linked to the federal platform laboratories. The second panel, distributed in June 2021, was set up for a two-part EQA scheme, namely a mandatory qPCR analysis and an optional SARS-CoV-2 mutation PCR analysis, the latter being not obliged since not all federal platform laboratories performed this test. The EQA panel consisted of seven samples with viral loads ranging from 873 to 2.9 × 10^6^ RNA copies/mL (three wild type SARS-CoV-2 strains, two Alpha, one Beta and one Gamma variants), supplemented with three SARS-CoV-2 negative samples. Seven laboratories (7/8; 88%) obtained the maximum score for the mandatory qualitative detection of the SARS-CoV-2 virus, while one laboratory reported a false positive result. Results were reported within 16 h for seven laboratories, however, one laboratory reported their results five hours outside the predetermined TAT. The optional SARS-CoV-2 mutation PCR analysis was performed by six laboratories of which five laboratories (5/6; 83%) correctly identified the SARS-CoV-2 variant for all samples tested, one laboratory reported two erroneous results. For each incorrectly reported result or TAT-deviation, laboratories were required to perform a root-cause analysis and integrate it in a corrective and preventive actions (CAPA)-plan, which was critically reviewed by Sciensano and the NRC during the lab’s following Q-meeting.

In addition to the EQA panels distributed by the NRC and Sciensano, all federal platform laboratories were obliged to participate to the external QC programme for SARS-CoV-2 organised by QCMD [24]. The first round, consisting of five samples with a viral load varying between 100 and 1.35 × 10^4^ RNA copies/mL, was distributed in May 2021, for which four laboratories (4/8; 50%) obtained the maximum score. Due to wrong contact details, one laboratory did not receive the QCMD-panel and was therefore not able to perform the analysis. Two other laboratories achieved a score of 4/5 and 2/5, as they were not able to detect samples below a viral load of 100 and 871 RNA copies/mL respectively. One laboratory was only able to report one correct result as they reported samples with a viral load below 1412 RNA copies/mL as negative. Since the extraction kit already in use from the start-up of the federal platform laboratories proved not sensitive enough over time, an extraction kit with higher sensitivity was selected through a new public tender in May 2021. The federal platform laboratories switched to the new extraction kit one at a time for logistics reasons, therefore not all laboratories were using the same workflow when processing the first round of QCMD samples. The three laboratories that incorrectly reported one to four samples negative, repeated the analysis after the implementation of the new extraction kit and were able to correctly detect all five samples. In August 2021, the laboratories received a second QCMD panel with viral loads ranging from 2.7 × 10^3^ to 8.9 × 10^4^ RNA copies/mL, all laboratories (8/8; 100%) reported results consistent with the foreseen outcome.

The federal platform laboratories collectively tested more than five million clinical samples over a course of two years (Fig. 2). They served as a buffer capacity and during peak periods, more than 20% of the total number of PCR tests were performed through this platform. Since the SC laboratories of the national testing platform were considered an extension of the NRC laboratory, the NRC was responsible for monitoring quality and following up on NCs and complaints. As described above, the federal platform laboratories were working under the QMS and direct supervision of their partner clinical laboratory, which improved the follow-up. However, the NRC and Sciensano did continue to monitor the quality of generated results through close contact with the laboratories and EQAs.

## Extending routine diagnostics for SARS-CoV-2 to more detailed reporting and detection of variants of concern

### Semi-quantitative reporting of SARS-CoV-2 PCR results

Over the course of the pandemic, the question arose to report results in a semi-quantitative manner in order to guide clinicians in their decision making, since the strength of the signal measured by PCR could assist in detailing the stage of infection and interpreting a positive test result in the context of clinical (onset of symptoms or timing of high-risk contact), serological evidence and/or a previous PCR test result. Since Cq values can widely vary across different methods and laboratories, four categories of positivity were defined based on absolute vial load quantification (copies/mL): very strongly positive (≥ 10^7^ RNA copies/mL), strongly positive (≥ 10^5^—< 10^7^ RNA copies/mL), moderate positive (≥ 10^3^—< 10^5^ RNA copies/mL) and weakly positive (< 10^3^ RNA copies/mL) [30]. A large volume of heat-inactivated SARS-CoV-2 control material was prepared in a high concentration to support all Belgian laboratories that performed SARS-CoV-2 assays in routine diagnostics. Each laboratory received instructions to prepare a standard curve using the pre-quantified stock and were asked to report the results to the NRC. For each individual PCR assay, the mean Cq value or equivalent metric, with corresponding SD was calculated per target gene, followed by the calculation of the mean Cq value for the three threshold concentrations (10^7^, 10^5^ and 10^3^ RNA copies/mL) to be able to translate conclusions into the four proposed categories of positivity. In total, a summary was provided for 17 different RT-PCR kits and communicated as such to all participating laboratories to support the harmonisation of semi-quantitative reporting of SARS-CoV-2 RT-PCR results [14].

### Rapid identification of variants of concern

The emergence of new SARS-CoV-2 variants became worrisome as the pandemic progressed. Alpha or B.1.1.7 was defined as the first VOC, which was first detected in the United Kingdom, and showed to impact the performance of certain diagnostic PCR kits which targeted the S-gene. When using the TaqPath COVID-19 CE-IVD RT-PCR assay (Thermo Fisher Scientific, Waltham, Massachusetts, United States), targeting the SARS-CoV-2 N-gene, ORF1ab and S-gene, the 69–70 deletion of amino acids located in the N-terminal domain of the spike S1 fragment caused a drop-out of the S-gene signal due to inability of the primer panel to anneal to the S-gene when characterised by the two site deletion, called S-gene target failure (SGTF) [31, 32]. Since all federal testing platform laboratories made use of this qPCR assay and analysed their PCR data using the same software, which centralised all results, their test results were used to monitor the first weeks of the emergence of several VOCs, more particularly Alpha, Omicron BA.1, BA.4 and BA.5 since they are all characterised by the presence of the 69–70 deletion [33]. Considering a large number of tests was being processed on a daily basis across these eight laboratories, information with respect to the circulation of these VOCs was provided much faster than any other typing method (SARS-CoV-2 mutation PCR or WGS) is technically able to do.

However, as not all VOCs are characterised by SGTF and a wide arsenal of qPCR assays is being used in the context of routine diagnostics throughout the country, more and more laboratories started to develop or implement LDT or commercially available mutation PCR assays to rapidly detect the presence of biologically relevant amino acid mutations located in the receptor-binding domain of the S-gene, with a first focus on mutations N501Y, E484K, K417T and K417N as those allowed to detect and discriminate the co-circulating VOCs Alpha, Beta and Gamma. At the NRC, the TaqMan SARS-CoV-2 mutation assays (Thermo Fisher Scientific, Waltham, Massachusetts, United States) were validated for the rapid detection and discrimination of SARS-CoV-2 VOCs, designing and optimising the panel of biologically relevant mutations in the spike protein along the course of the pandemic. Depending on the (co-)circulation of VOCs, a different set of target mutations was selected and evaluated using a broad panel of strains characterised by WGS [33–36]. In short, RNA is first reverse transcribed and amplified in a one-step RT-PCR reaction using sequence specific primers to amplify the region of interest. The reverse primer in the assay is used to initiate reverse transcription of the SARS-CoV-2 genomic RNA sequences. Each assay allows the detection of single mutations using two or three TaqMan minor groove binder probes with nonfluorescent quenchers, followed by cluster plot analysis to distinguish the detection of the mutation or the reference sequence. To assist the technical evaluation of such mutation PCR assays in clinical laboratories and in the federal platform laboratories, positive control material of different VOCs was prepared and distributed by the NRC after characterising the amino acid mutations and deletions in the SARS-CoV-2 genome for each stock using WGS [33–36]. Overall, 21 laboratories requested and received control material of one or several VOC(s).

### SARS-CoV-2 genomic surveillance

In the first year of the pandemic, genomic surveillance for SARS-CoV-2 was carried out by experienced scientists from a few universities in Belgium, including UZ/KU Leuven. At the start of 2021, a nationwide genomic surveillance consortium consisting of 17 laboratories was established in Belgium to markedly increase the country’s genomic sequencing efforts in terms of intensity and representativeness [13]. Per province or region, one or more sequencing laboratories were selected to ensure sufficient sequencing capacity. A total of 13 laboratories were selected and signed a convention with RIZIV-INAMI to be officially recognised as SARS-CoV-2 sequencing laboratory, with each laboratory required to demonstrate an accredited next generation sequencing (NGS) activity, not limited to molecular microbiology, in their organisation. Eleven laboratories were already performing NGS activities under accreditation, while two laboratories had to go through the BELAC accreditation process in order to be officially recognised [37]. These network laboratories receive samples from a wide variety of clinical laboratories across Belgium according to defined indications for sequencing [38–41] and analyse samples using different sequencing protocols (Illumina vs Oxford Nanopore Technologies), as described in Cuypers et al. [13]. Coordination of the consortium is carried out by the NRC in collaboration with Sciensano, by providing support to the laboratories of the consortium through training, organising weekly to monthly meetings, managing the surveillance program, organising quality assurance initiatives and publishing weekly reports to inform the broad public of the consortium’s output [42]. To create an overview of protocols the laboratories were using, and to discuss and evaluate quality metrics already in place, weekly Q-meetings were organised in the first two months after the launch of the consortium. Therefore, all laboratories provided detailed descriptions regarding their wet-lab procedures, bioinformatics and reporting, after which the NRC mapped the entire process in detail (e.g., primers, controls, bioinformatics pipelines, read mapping, classification, …) for each laboratory, which was thoroughly discussed with all partners of the consortium. In addition, the potential use of backup reagents and protocols were reviewed during these meetings.

Since no official EQA existed for SARS-CoV-2 WGS at the time of the start of the consortium, various cross-validation rounds were organised to follow up on quality assurance. Participation to the EQA is mandatory for all laboratories that signed the convention with RIZIV-INAMI (13 out of 17 network laboratories) [37]. In 2021, the NRC organised three cross-validation rounds during the months February, May and October. For each round, participating laboratories contributed a sufficient volume of leftover sample material for which they obtained high-quality WGS information. These samples were used to constitute panels, consisting of three samples for each laboratory, of a variety of SARS-CoV-2 variants as well as different transport media. Samples were blinded and distributed among the participating laboratories, with for each sample a cross-validation being performed by two or three laboratories. FASTA consensus sequences of the submitting laboratory as well as of the one or two receiving laboratories were compared to each other on the same date using the Nextclade [15, 16] and Pangolin webtools [17, 18] (since these classification tools are often updated). Detected mutations and deletions were reviewed and compiled in a report per laboratory, together with the detected strain, achieved coverage, used methods and pipelines. Additionally, a final summary report was written to communicate to all participating laboratories. Depending on the results (Table 3), details on scoring regarding classification and TAT and, if applicable, accompanying action points were communicated [13]. In 2022, an additional cross-validation round was organised towards the second half of the year (results not shown).

**Table 3:** WGS cross-validation results for all three rounds organised in 2021. Results are considered correct if the receiving laboratory reports results in concordance with the sending laboratory. Lab 15, not part of the convention, did not participate to the third cross-validation round, organised in October 2021. Lab 6 was unable to obtain a result for 1/3 cross-validation samples for the first two rounds. In the first case they voluntarily tested a new panel for which they obtained the maximum score; as an action for the second round, the failed sample was sequenced by the NRC, who was also unable to type the sample. Therefore, it was assumed that RNA degradation had occurred during transport from the sending laboratory to the NRC. For round one, lab 17 reported two samples as ‘undetermined’ due to low cDNA amplification. For the second round, lab 10 could not obtain a result for one cross-validation sample. The laboratory indicated this problem occurred when UTM samples were extracted using the extraction kit from the federal platform laboratories (note that federal platform laboratories only routinely test samples taken in virus inactivating medium). Lab 9 was unable to report a result for two cross-validation samples due to low RNA concentrations

	Lab 1	Lab 2	Lab 3	Lab 4	Lab 5	Lab 6	Lab 7	Lab 8	Lab 9	Lab 10	Lab 11	Lab 12	Lab 13	Lab 14	Lab 15	Lab 16	Lab 17
*Round 1*	3/3	3/3	3/3	3/3	3/3	**2/3**	3/3	3/3	3/3	3/3	3/3	3/3	3/3	3/3	3/3	3/3	**1/3**
*Round 2*	3/3	3/3	3/3	3/3	3/3	**2/3**	3/3	3/3	3/3	**2/3**	3/3	3/3	3/3	3/3	3/3	3/3	3/3
*Round 3*	3/3	3/3	3/3	3/3	3/3	3/3	3/3	3/3	**1/3**	3/3	3/3	3/3	3/3	3/3		3/3	3/3

## Evaluation of sample collection material and available kits on the market

Due to the scarcity of reagents and sample collection materials in April 2020, the NRC was requested by the governments task force to evaluate alternative transport media that were available on the market and whose purchase could be assured. For each sample collection kit, a technical evaluation was performed along with an evaluation of the corresponding transport medium, for which SARS-CoV-2 RNA stability was evaluated over a time period of five days at room temperature. In total, 16 different sample collection kits and accompanying transport media were evaluated, of which nine received a positive advice. For seven other sampling kits, a negative advice was given either due to poor quality of the collection material, significant changes in the Cq values of the exogenous internal control or due to unacceptable RNA stability.

In preparation for potential reagent shortages, the NRC conducted a precautionary evaluation of generic reagents during the summer of 2020. For all extraction, PCR and S2R systems used in the clinical laboratories, a distinction was made between closed and open systems and, for the latter, a search for potentially compatible reagents was carried out. To perform a validation of these potentially compatible reagents, suppliers were contacted to obtain reagents. Unfortunately, due to a sudden rise in the demand for SARS-CoV-2 testing, only one clinical laboratory expressed interest and performed this validation. The tested PCR kit met all validation criteria and could be implemented in routine diagnostics.

Along the summer period of 2020, the government purchased pre-barcoded standardised sample collection material which contained the virus inactivating transport medium DNA/RNA Shield (Zymo Reagents) containing guanidinium thiocyanate (GTC). The main objective was to optimise exchange of samples between clinical laboratories and transfer of samples to the supporting laboratories of the federal platform, which would launch from October 2020 on. Each federal platform laboratory would exclusively process samples collected in DNA/RNA Shield and additionally, would be able to increase their testing capacity up to 7,000 samples per day, therefore allowing samples to be transferred to or between federal testing laboratories without significantly impacting TAT. Clinical laboratories could forward their overflow samples to a federal platform laboratory only if the samples had been collected in DNA/RNA Shield. Therefore, the NRC aided all Belgian clinical laboratories with the validation of this GTC-transport medium. To identify which laboratories wished to perform the validation and on which extraction- and/or PCR-platform(s), a survey was distributed. At the time of the survey, 93 clinical laboratories were eligible to conduct diagnostic SARS-CoV-2 PCR-testing, of which 81 laboratories (87.1%) responded to the survey. Fourteen laboratories (14/81 or 17.3%) indicated that they either did not feel the need to send samples to the federal platform laboratories, their platform(s) were not compatible with the GTC-medium or they did not have sufficient time or reagents to perform the validation. Sixty-seven laboratories (67/81 or 82.7%) expressed interest in evaluating the standardised collection material and thus received material via the NRC to perform a validation in accordance with their own procedures. Eleven laboratories (11/67) did not submit their results, even though they had received the necessary material. Eventually, 56 laboratories performed the validation experiments, of which 45 laboratories (45/56 or 80.4%) successfully validated one or more platforms in their laboratory, the platforms of the remaining laboratories (11/56 or 19.6%) did not turn out to be compatible with the GTC-medium, either due to inhibition or due to strong shifts in Cq values. The NRC consolidated and shared all results, which were discussed during the following COVID-19 testing information sessions (see Table 2). From then on, laboratories were able to order the standardised sample collection material for use in their own laboratory or to send overflow samples to the federal platform laboratories.

## Conclusions

Thanks to a nationwide collaboration between the NRC UZ/KU Leuven, Sciensano, the Belgian government, the newly established testing platforms and all clinical laboratories, Belgium effectively responded to the high demand for COVID-19 testing during the ongoing pandemic. Initially, diagnostic testing for SARS-CoV-2 was solely conducted at the NRC. However, clinical laboratories swiftly implemented SARS-CoV-2 diagnostic assays with the support and technical expertise of the NRC. Nonetheless, this proved insufficient to meet the testing demand during Belgium’s initial wave of the epidemic. To facilitate the rapid expansion of testing capacity, the national testing platform was established as an extension of the NRC laboratory. The SC laboratories within the national testing platform were continually monitored through a proficiency testing program organised by the NRC. Subsequently, the federal platform laboratories assumed responsibility for performing SARS-CoV-2 PCR tests under the supervision and QMS of their partner clinical laboratories. Nevertheless, the NRC and Sciensano maintained close contact with the laboratories and organised EQA’s to ensure the quality of test results. In an effort to harmonise the reporting of SARS-CoV-2 PCR test results at a national level, laboratories adopted a semi-quantitative reporting approach. This method provides clinicians with more detailed information on the stage of infection, indirectly correlated with infectivity. When the emergence of new VOCs raised alarm, the NRC supported laboratories interested in implementing SARS-CoV-2 mutation PCR assays by providing control material from different VOCs. Additionally, the NRC played a key role in establishing and coordinating a WGS consortium to monitor all circulating virus strains. Despite the rapid outbreak of this pandemic, the limited knowledge on SARS-CoV-2 detection in the laboratories and the limited experience in rapid upscaling of molecular test capacity, all Belgian laboratories successfully conducted large scale SARS-CoV-2 PCR testing.

## Data Availability

Datasets used and/or analysed in this study are available from the corresponding author on reasonable request.

## Abbreviations

BELAC: Belgian accreditation body
CAPA: corrective and preventive actions
CMIA: chemiluminescent microparticle immunoassay
CMV: cytomegalovirus
COVID-19: Coronavirus disease 2019
EQA: external quality assurance
EUA: emergency use authorization
FAMHP: Federal Agency for Medicines and Health Products
FDA: Food and Drug Administration
GTC: guanidinium thiocyanate
HCoV: human coronavirus
HSV: herpes simplex virus
IC: internal control
iQC: internal quality control
IVD: in vitro diagnostic medical device
LDT: lab-developed test
LOPOS: weakly positive
N/A: not applicable
NA: nucleic acid
NC: non-conformity
NGS: next generation sequencing
NRC: national reference centre
ONT: Oxford nanopore technologies
PIV: Parainfluenza virus
POS: positive
QC: quality control
QCMD: Quality Control for Molecular Diagnostics
QMS: quality management system
qPCR: quantitative polymerase chain reaction
RSV: respiratory syncytial virus
RUO: research use only
S2R: sample-to-result
SARS-CoV-2: severe acute respiratory syndrome coronavirus 2
SC: subcontracting
SGTF: S-gene target failure
TAT: turn-around time
TMA: transcription-mediated amplification
TMO: Thermo Fisher Scientific
UZ Leuven: University Hospitals Leuven
VOC: variant of concern
WGS: whole genome sequencing
